# Decentralized clinical trials (DCTs): A few ethical considerations

**DOI:** 10.3389/fpubh.2022.1081150

**Published:** 2022-12-15

**Authors:** Carlo Petrini, Chiara Mannelli, Luciana Riva, Sabina Gainotti, Gualberto Gussoni

**Affiliations:** ^1^Bioethics Unit, Istituto Superiore di Sanità, Rome, Italy; ^2^Clinical Research Department, FADOI Research Centre, Milan, Italy

**Keywords:** DCTs, Research Ethics, Bioethics, healthcare, digitalization

## Abstract

Decentralized clinical trials (DCTs) are studies in which the need for patients to physically access hospital-based trial sites is reduced or eliminated. The CoViD-19 pandemic has caused a significant increase in DCT: a survey shows that 76% of pharmaceutical companies, device manufacturers, and Contract Research Organizations adopted decentralized techniques during the early phase of the pandemic. The implementation of DCTs relies on the use of digital tools such as e-consent, apps, wearable devices, Electronic Patient-Reported Outcomes (ePRO), telemedicine, as well as on moving trial activities to the patient's home (e.g., drug delivery) or to local healthcare settings (i.e., community-based diagnosis and care facilities). DCTs adapt to patients' routines, allow patients to participate regardless of where they live by removing logistical barriers, offer better access to the study and the investigational product, and permit the inclusion of more diverse and more representative populations. The feasibility and quality of DCTs depends on several requirements including dedicated infrastructures and staff, an adequate regulatory framework, and partnerships between research sites, patients and sponsors. The evaluation of Ethics Committees (ECs) is crucial to the process of innovating and digitalizing clinical trials: adequate assessment tools and a suitable regulatory framework are needed for evaluation by ECs. DCTs also raise issues, many of which are of considerable ethical significance. These include the implications for the relationship between patients and healthcare staff, for the social dimension of the patient, for data integrity (at the source, during transmission, in the analysis phase), for personal data protection, and for the possible risks to health and safety. Despite their considerable growth, DCTs have only received little attention from bioethicists. This paper offers a review on some ethical implications and requirements of DCTs in order to encourage further ethical reflection on this rapidly emerging field.

## Introduction

Decentralized clinical trials^1^ (DCTs) make use of digital technologies and other methods to enable access of patients to clinical research, remote data collection and monitoring, and communication between the investigators and participating subjects.

In a DCT, enrolled patients are no longer required to frequently travel to a healthcare facility in order to participate in the trial, as they are able to take part from their normal living environment. The center of gravity of the trial therefore shifts from the study site (i.e., the hospital) to the patient's home. Thus, DCTs can adapt to patients' routines and allow them to participate regardless of their geographical position.

DCTs can include the direct delivery of investigational medicinal products (IMP) to participating subjects, laboratory examinations and/or instrumental tests carried out in centers other than the trial site and close to the patient's home, and home visits by healthcare professionals. This study model typically involves use of Internet, smartphones and their applications, telemedicine platforms, social media and similar technologies at different stages of the trial (patients' enrolment and consent, clinical checks, remote data collection, monitoring and source data verification).

In DCTs, remote data collection can be active or passive. When it is active, the patient is required to enter data using one or more devices, whereas when it is passive the data are logged by the device/s used in the study (e.g., wearables or sensors) without active intervention by the patient. In both cases, patient involvement in data collection may actually be more active than with conventional participation at a healthcare facility. Furthermore, by means of electronic instruments, DCTs allow constant contact between the patient and research staff.

DCTs are not an all-or-nothing method, as the decentralization can be of varying degrees. The use of technology does not exclude personal interaction or the possibility of the patient traveling to a healthcare facility and participating in the trial under certain circumstances. More specifically, a DCT may include procedures that cannot be carried out in a home environment. Many DCTs are therefore in hybrid form, combining home-based, traditional on-site visits, and study procedures.

DCTs are especially useful in cases that make travel difficult for the patient, either for clinical conditions (e.g., neuromuscular diseases), or logistical barriers, when research sites are far from patient's home (as often occurs in case of rare diseases). Decentralized studies are particularly suitable for low- to medium-complexity conditions, and for studies that are not excessively long.

In light of the above, it should be noted that currently, in many cases, DCTs do not replace conventional trials. Rather, they are supplementary to them.

The therapeutic areas for which DCTs are most readily applicable are those in which telemedicine is most advanced like diabetes, neurorehabilitation, cardiovascular diseases, pulmonary diseases and, more recently, COVID-19.

One of the main challenges in the implementation of DCTs (in Europe and worldwide) at the current time regards the fact that the existing regulatory frameworks were devised with conventional clinical trials in mind. Besides, there are still very few documents and guidelines on the planning, design and evaluation of DCTs and decentralized methods (1), and this is in some ways surprising since DCTs are not absolutely a novel mode.

Indeed, the earliest studies on the feasibility of “Internet trials” date back to 2003. Since then, there has been a continuous crescendo, for example the first “Trial over the Internet” was patented in the USA in 2007. In 2011, Pfizer conducted the first fully-decentralized randomized study titled “Research on Electronic Monitoring of Overactive Bladder Treatment Experience, REMOTE” (2, 3), the results of which were published in 2014. In this trial, the Internet was used for subject enrolment, the administration of online screening questionnaires and provision of electronic outcomes diaries, and the investigational medicinal product was delivered to the patients' homes.

Over the last decade, all major pharmaceutical companies have conducted DCTs. According to a survey carried out by the consulting company McKinsey in December 2019, immediately before the pandemic, 38% of representatives of the pharmaceutical industry and contract research organizations (CROs) anticipated that the majority of their activities would be comprised of “virtual” studies and 48% anticipated conducting trials in which most of the activities would be carried out at patients' homes. When McKinsey asked the same questions 1 year later, the answers were 100 and 89%, respectively (4).

It should therefore be pointed out that the COVID-19 pandemic has stimulated a considerable increase in DCTs: a survey conducted by Oracle (5) showed that, already in the 1st year of the pandemic, 76% of pharmaceutical companies, device manufacturers and CROs had adopted decentralized techniques. Of these, 7% used fully decentralized methods. Actually, COVID-19 has provided a significant proof of concept (PoC) regarding clinical trials in a context of emergency and, in particular, on the integration of decentralized approaches.

In order to support the on-going process, in March 2020 the FDA issued in the United States specific operational guidelines covering many of the challenges posed by the decentralization of activities in clinical studies, with its “Guidance for Industry, Investigators, and Institutional Review Boards” (6).

On 4 February 2021, the European Commission published the fourth version of its guidelines on the management of clinical studies during the COVID-19 pandemic, i.e. “Guidance on the Management of Clinical Trials during the COVID-19 (Coronavirus) Pandemic.” Despite the temporary nature of these guidelines, which were designed for the management of clinical trials during the health emergency, they contain key information for the implementation of DCTs. Moreover, they include the authorization of procedures such as the delivery of investigational products at patient's home, home-based visits, use of community-based diagnostic facilities, and remote monitoring of collected data / source data verification (7). Now the question is whether, which and how those methods authorized by the central European and local authorities during the emergency will pass from derogation to rule. In certain European countries, in recent years, the competent institutions have started to deal with the matter starting from the local regulatory framework, in order to provide guidance to investigators and sponsors (8, 9).

It is crucial to carry out feasibility studies in order to identify the opportunities and the challenges from a regulatory standpoint and to favor the authorization and implementation of DCTs (10).

The opportunities appear to be numerous. It has been suggested that DCT approaches can be justified and particularly suitable for trials with chronic diseases, rare diseases, immobile participants, self-administrable IMP, lower safety risk profile, and confirmatory clinical trials. Particularly in rare disease studies, the changes necessitated by the COVID-19 pandemic have provided an opportunity to become a standard approach. Although some DCT projects were developed even earlier in this area, during COVID-19 they forcibly entered clinical practice offering advantages in terms of patient burden, practicality, inclusion and data quality (11).

However, not all clinical trials are suitable for decentralization and hybrid solutions appear as the more reasonable scenario in the very majority of cases. Future research will be needed to demonstrate, for example, whether studies on DCTs or hybrid DTCs are particularly suitable for carrying out prevention or screening studies compared to treatment clinical studies.

## The risks and benefits of DCTs

The possibility of decentralizing studies affords a number of opportunities, with ethical and clinical implications (12–16). The potential advantages of DCTs include:

- The possibility of enrolling subjects who are unlikely to be able to take part in conventional trials, because their home is a long way from a healthcare facility, or because of physical difficulties in reaching the facility. Facilitated access allows a higher number of patients to be eligible for participation. This aspect is particularly important, especially in research on rare diseases, because it favors inclusion, and improves the representativeness and generalisability of the results.
- More convenient conditions for subjects, with less avoidable discomfort and suffering, in particular for frail subjects. DCTs do away with waiting times, contact with the suffering of other patients, in some cases hospitalization, possible exposure to pathogens in hospital settings that can cause complications.
- Greater autonomy for the participating subject, who can remain at home at least for part of the study procedures.
- Greater convenience for families and caregivers.
- The possibility of collecting “real-time” and “real-world data,” in the subjects' usual living environment and therefore avoiding potential bias resulting from assessments performed in *ad hoc* facilities.
- The possibility of evaluating endpoints difficult to measure with conventional studies, thanks to the ways in which the data can be collected.
- Time-saving.
- Cost-saving.

Although some studies show an increase in patient retention rates in DCTs and better compliance with procedures than in conventional trials (due to the home setting, use of electronic reminders, an overall less burdensome participation etc.), there is no full consensus regarding these aspects in the literature (17, 18).

Nevertheless, despite considering the significant benefits decentralized trials can offer, it is also necessary to mention disadvantages (some of them may occur in CTs as well). Potential barriers, limitations and risks associated with the implementation of DCTs (19–24), include:

- Potential amplification of inequalities. Groups with reduced access to technologies could be penalized. This aspect should be considered both in relation to the ability to use devices and the availability of the equipment required for connection, i.e., access to a stable connection (which may depend on both economic and geographical factors) and of supporting devices.
- Partial application (DCTs are not suitable for all medical conditions).
- Remote data collection can favor quality, thanks to automation of the processes involved. However, the quality of collection can be jeopardized as it takes place in a less “protected” setting than a research facility. This may lead to the risk of technical failures when digital devices are used. Therefore, deterioration in data quality at the source and during transmission are possible.
- Risks regarding the validity and reliability of the data collected. One example is the “6-min walk test” used to assess the effects of treatments aimed at improving walking capacity in patients with peripheral artery disease. In order for the data to be reliable, the test must be performed by making the patient walk on a rigid and flat surface, and of accurately documented length. However, when the test is carried out by a patient at home, it may be troublesome to ensure that the surface meets the requirements, is obstacle-free and precisely measured (25). This inconsistency could have an impact on the reliability of the data. Although mistakes and inaccuracy may occur in conventional trials as well, factors that may jeopardize validity and reliability in DCTs should be properly identified and addressed.
- A methodological bias may arise from the combined use of clinical measurements performed in a hospital or home setting. A typical example is that of arterial blood pressure measurement.
- Some clinical checks may be less accurate if conducted remotely. This can lead to potential issues for the wellbeing and safety of patients.
- Risks regarding the protection of personal data, also due to the increased number of actors involved (e.g., couriers for delivery of the investigational product, providers for home assistance and digital services, etc).
- Data breach risks.
- Risk of weakening the physician-patient relationship.
- Potential isolation of the trial subject, who does not have opportunities to meet and share experiences with other patients taking part in the same study.

## Some requirements

### Information and consent

Given their nature, DCTs often involve the use of e-consent of various forms.

E-consent has advantages over the conventional paper form, for example it can be filed easily, retrieved rapidly, updated readily and promptly shared amongst the staff involved. Among relevant aspects, it is important to ensure that the systems used for e-consent have proportionate security levels, and safeguards regarding confidentiality are in place.

Special care must be dedicated to the clarity and completeness of the information provided to the patient. In the case of fully-digital consent, the validity of the signature must be guaranteed from a legal perspective as well. It must be borne in mind that electronic signatures, particularly Advanced Electronic Signatures (AdESs) require identification and registration procedures that could be complicated for some subjects: this could hamper, or even preclude, the access of certain population groups.

If the personal relationship with the healthcare professionals is important in the information and conventional consent procedure, it is even more so in DCTs. Indeed, as DCTs are conducted remotely, personal contacts are infrequent (or completely absent): it is therefore important to provide chances for direct exchange and communication during the initial stage and whenever the need arises. In this perspective, face-to-face communication should take place between the investigator and the potential trial participant. If this discussion takes place in a digital / virtual mode, this should be generally performed in real time where the parties are able to see and communicate with each other *via* audio and video, and to ask questions.

### Access

DCTs can increase the number of individuals eligible for a trial by removing the logistical and geographical barriers but, at the same time, they can increase inequalities in access, penalizing individuals who do not possess the technologies or the skills required. The availability of technologies should not constitute an exclusion criterion and the necessary equipment should be provided by the sponsor.

Participants and, if necessary, also their caregivers, should be provided not only with initial training on using the devices, but also with on-going support throughout the progress/evolution/unrolling of the DCT.

### Data collected, transmitted, and analyzed

In general, special care must be taken when applying the basic criteria that pertain to all data processing:

- Lawfulness, fairness, and transparency. Data must be processed lawfully, fairly, and in a transparent manner in relation to the data subject.
- Restriction of the purpose: data must be processed for specified, explicit, and legitimate purposes. They must also be processed in a manner that is compatible with such purposes.
- Minimization: personal data must be adequate, relevant and restricted to the purposes for which they were collected.
- Accuracy and updating: data must be accurate and up-to-date. There must be procedures in place for the timely correction or erasure of inaccurate data.
- Restriction of storage: data must be stored in a form that permits the identification of the data subjects only for as long as is strictly necessary to fulfill the purposes for which they were processed, unless that patient has explicitly consented to reuse the data for future research.
- Integrity and confidentiality: personal data must be guaranteed adequate security.
- Accountability: the controller must ensure that the data are processed in an appropriate manner.

Data must be: Attributable, Legible, Contemporaneous (i.e., recorded at the time the activities are carried out), Original (or true to the original), Accurate (ALCOA) (26).

In order to allow the reuse of existing data and avoid useless duplications, data should also be made Findable, Accessible, Interoperable and Reusable (FAIR) (27).

The accuracy of data recording is particularly important in the case of active data collection by the patient. Therefore, the subjects taking part in trials must be given adequate instructions on this matter.

There must be commensurate procedures in place to ensure the integrity of the data during their transmission and management. Special attention must be given to the fact that the data stored on personal devices can be easily linked to other personal data (contacts, location, microphone, video camera, purchases, etc.). Therefore, there must be adequate procedures in place to guarantee the effective protection of all personal data. The risks of unintentional data disclosure or deliberate breach of privacy go well beyond the scope of the DCTs. For example, health-related information can result in discrimination in the workplace. In order to reduce this kind of risk, it may be useful to use distributed ledgers, decentralized databases and blockchain technology (28, 29).

The final use of the data must also be strictly governed: DCTs favor the collection of a multitude of real-world data, which in some cases go beyond the scope of the study. Therefore, it is necessary to prevent their use in contexts other than those envisaged by the study. It is also necessary to clearly establish which data may be used after the end of the DCT. Patients must be adequately informed of this possibility and given the chance to grant or refuse their consent to such use.

### Study protocol flexibility

Preferences vary from one person to another. For example, some may prefer direct personal interactions, without the mediation of technology.

Flexible research programmes can make it possible to not overlook differences in personal preferences. To this end, it would be useful to give patients the possibility to provide regular feedback on their experience regarding the trial. However, flexibility may also introduce the risk of methodological biases (see what previously reported on arterial blood pressure).

### Provision of the IMP

In planning a clinical trial, the sponsor and investigator may consider whether the IMP is suitable for administration at home, and if the appropriate storage conditions of the IMP can be met. In DCTs, the medicinal product can be delivered to the subject's home, usually by courier, under supervision and responsibility of the pharmacy of the healthcare facility and the investigator. In addition to rigorous protection of privacy, the distribution system must guarantee quality and efficiency, particularly for medicinal products requiring special storage and transportation conditions (for instance: maintenance of the cold chain).

### Return of result

Patients generally wish to know the results of the clinical investigations in which they are involved as soon as possible. In DCTs, given the way the studies are conducted, patients may be even more eager to find out the results quickly.

Among other aspects, special attention must be paid to the occurring of any incidental findings, in other words, unexpected results that are not related to the study and are not intentionally sought. In the case of incidental findings that are clinically relevant (for prevention and therapy) and actionable, it is the physician's duty not to overlook them: therefore, precise procedures must be adopted for the management of any incidental findings (30–32).

## Discussion

The DCT approval process deserves special attention, making the role of Ethics Committees (ECs) crucial.

The procedures for conducting DCTs are such that the current regulatory framework may be only partially adequate. There are no detailed documents or reference standards concerning the role of ECs in the oversight and evaluation of DCTs (33). These bodies may encounter difficulties when reviewing studies that involve significant complexities due to the innovative approaches employed. Information on the decentralized activities should therefore be clear and justified on a case-by-case basis in the clinical trial protocol (34). In a simulated survey on members of European ECs called on to review a DCT protocol, it was observed that the quality, safety, and organization of the DCT were perceived as being more problematic than those of conventional clinical studies. For instance, the members expressed concerns regarding the validity and accuracy of the data, in case the participating subjects were responsible for measuring and inputting them (1). Although the criteria used by ECs when analyzing a decentralized clinical study are the same as those for conventional studies, the application of such criteria to the specific cases can be more complex. Moreover, EC members may require supplementary information in order to consider, for example, whether the procedures for implementing the electronic informed consent process are suited to guaranteeing a true personal data communication, comprehension, and protection process.

Aspects examined by ECs when reviewing new studies must be considered in the light of the DCTs as well. This scenario may be troublesome because of the numerous peculiarities DCTs show. One example regards the assessment of site suitability. DCTs are coordinated by trial sites, whose suitability can be assessed using the conventional criteria. However, DCTs are conducted at the subjects' home, which makes it difficult, if not impossible, to guarantee *a priori* that each home is fully suited to the conduct of the DCT in question.

Another example regards the way in which devices are used. Most (but not necessarily all) the devices used in DCTs are classified as medical devices. The medical devices must be marked pursuant to regulations and used in compliance with their intended use. If this were not the case, the DCT would qualify as a clinical investigation on a medical device. This actually creates a intertwining between the regulations governing clinical trials on medicinal products and those on medical devices that is often difficult to manage, especially for ECs.

More generally, adequate guidelines, recommendations and regulations must be adopted in order to favor harmonization of both DCT review and authorization procedures and foster virtuous implementation of these trials.

At European level, an in-depth review of the ethical and legal framework is essential for establishing how the existing definitions and conceptual rules for clinical trials are applicable to the decentralized activities of DCTs. As digital technologies gradually become more extensively incorporated into clinical trials, the EU regulatory framework for DCTs/hybrid trials will have to evolve and the Good Clinical Practice (GCP) protocols will have to be modernized. Modernizing GCP regulatory supervision in order to enable decentralized clinical study models is currently an objective for the European institutions (35).

The need for homogeneous safety standards that guarantee patients a level of protection not lower than that adopted for conventional trials, is particularly important: the fact that DCTs can allow real-time continuous monitoring is not, in itself, a guarantee of adequate protection. It is also necessary to adopt procedures that lead to timely intervention and, preferably, provide a remedy in the case of unforeseen circumstances, incidents and adverse events. This calls for effective e-health systems that are suited to the purpose, and above all a health organization that guarantees 24/7 surveillance and possible assistance.

E-health systems must, in any case, offer patients the possibility of direct contact with the healthcare facility and with the doctors and researchers conducting the trial. With a view to this, in many cases, hybrid DCTs are appropriate as they alternate procedures at the subject's home with procedures at the trial site.

Considering the growing number of DCTs and their challenging implementation, adequate training - both on the technical aspects, including digital skills, and on the ethical implications resulting from the decentralized methods - should be provided to stakeholders, namely:

- the healthcare personnel that design and conduct DCTs: all of them (including those that carry out home visits) must be technically and ethically skilled;
- the patients and their caregivers, who must be not only informed, but also trained;
- the EC members, so that they can play their responsibility for authorizing DCTs with competence and awareness.

The planning and conduct of DCTs involve particularly complex matters: partnerships between sponsors, study sites and patient advocacy groups must be favored to promote the best individual involvement of the patients themselves. General practitioners should also be involved.

DCTs must be planned and conducted maintaining the standards for the production of evidence commonly adopted by the scientific community: although for DCTs changes in the organizational, administrative, regulatory and operational conditions for the conduct are permitted, shortcuts and exceptions in the scientific method and rigor are not acceptable.

Groups that are unlikely to participate in DCTs because of digital divide (for example many elderly people) must be offered alternative options for trial participation, so that anyone who meets the eligibility criteria has the chance to take part. This is a major challenge for clinical research in the near future: combining and harmonizing the need for equity of access, the procedural flexibility offered by the availability of different methods of conducting studies, and the methodological rigor in the production of reliable scientific evidence.

Therefore, any decision to switch from a traditional trial to DCTs must be decided on a case-by-case basis: the elements of decentralization must be justified in relation to the characteristics of the study, and balancing improved access for patients, their safety, rights and dignity, with the quality of collected data. Respect for the person, his/her wellbeing and his/her central role must always come first, taking precedence over any procedural consideration regarding organization, quality, efficiency, effectiveness, and the progress of knowledge.

## Author contributions

CP wrote the initial draft of the manuscript with the contribution of CM. LR and SG contributed with insightful feedback and integrations. GG provided critical revision. All authors approved the final manuscript.

## Footnotes

^1^This paper only refers to DCTs on medicinal products.
